# Efficacy of bone stimulators in large-animal models and humans may be limited by weak electric fields reaching fracture

**DOI:** 10.1038/s41598-022-26215-w

**Published:** 2022-12-16

**Authors:** Nishant Verma, Todd Le, Jonah Mudge, Peter J. Nicksic, Lillian Xistris, Maisha Kasole, Andrew J. Shoffstall, Samuel O. Poore, Kip A. Ludwig, Aaron M. Dingle

**Affiliations:** 1 Department of Biomedical Engineering, University of Wisconsin-Madison, Madison, WI USA; 2 Wisconsin Institute for Translational Neuroengineering (WITNe), University of Wisconsin-Madison, Madison, WI USA; 3 Division of Plastic Surgery, School of Medicine and Public Health, University of Wisconsin-Madison, Madison, WI USA; 4 Department of Biomedical Engineering, Case Western Reserve University, Cleveland, OH USA; 5 Advanced Platform Technology Center, Louis Stokes Cleveland VA Medical Center, Cleveland, OH USA; 6 Department of Neurological Surgery, University of Wisconsin-Madison, Madison, WI USA

**Keywords:** Fracture repair, Biomedical engineering

## Abstract

Noninvasive electronic bone growth stimulators (EBGSs) have been in clinical use for decades. However, systematic reviews show inconsistent and limited clinical efficacy. Further, noninvasive EBGS studies in small animals, where the stimulation electrode is closer to the fracture site, have shown promising efficacy, which has not translated to large animals or humans. We propose that this is due to the weaker electric fields reaching the fracture site when scaling from small animals to large animals and humans. To address this gap, we measured the electric field strength reaching the bone during noninvasive EBGS therapy in human and sheep cadaver legs and in finite element method (FEM) models of human and sheep legs. During application of 1100 V/m with an external EBGS, only 21 V/m reached the fracture site in humans. Substantially weaker electric fields reached the fracture site during the later stages of healing and at increased bone depths. To augment the electric field strength reaching the fracture site during noninvasive EBGS therapy, we introduced the Injectrode, an injectable electrode that spans the distance between the bone and subcutaneous tissue. Our study lays the groundwork to improve the efficacy of noninvasive EBGSs by increasing the electric field strength reaching the fracture site.

## Introduction

Orthopedic trauma causes $256.4 billion per year of economic loss in the form of healthcare costs and time lost from work in the United States^1^. These huge costs have driven efforts to augment the well-studied process of fracture healing. Electrical stimulation is one method that has been explored to improve fracture healing. Fukada and Yusada discovered the piezoelectric property of bone—that bone generates endogenous electrical fields when put under mechanical stress—and its relationship to bone formation in 1953^2^. Since then, there have been multiple studies in in vitro, small animal, and large-animal models, as well as clinical studies, to explore the effects of electrical stimulation on an array of osseous injuries^3^. These studies have led to the approval of nine electronic bone growth stimulators (EBGSs) by the Food and Drug Administration (FDA) for use in the treatment of osseous nonunion^4^.

A recent survey study reported that only 32% of orthopedic traumatologists have ever used an EBGS, and the main reasons given were high costs and inconsistent clinical efficacy^5^. Direct current electrical stimulation (DCES) and capacitive coupling (CC) electrical stimulation are two popular forms of EBGSs. The clinical data suggest that direct current electrical stimulation methods appear to work better to promote bone healing than noninvasive stimulation methods but increase risk of infection^6^ and require a costly and complex invasive device that has a high rate of failure due to device-related complications. Capacitive coupling is typically a noninvasive method of electrical stimulation that involves electrodes placed on the skin on opposite sides of the osseous injury. Alternating current generates an electric field between the electrodes, but the penetration of this field into tissues is poor^7^, making this modality applicable to only superficial bones like the distal radius or tarsal bones.

The FDA Bone Stimulation workshop report^8^ reported that improvements in healing after fracture caused by the application of an electric field are believed to be due to the activation of voltage-gated calcium channels. The report summarized that the electric field causes voltage-gated Ca^2+^ channels in the cell walls of osteocytes to open, changing intercellular and cytosolic Ca^2+^ levels^8,9^. The change in intercellular and cytosolic Ca^2+^ levels triggers signaling molecules to promote osteoblastic differentiation and formation, thereby upregulating bone formation activity^8,10^. The putative mechanism of action depends critically on getting sufficient electric field strengths at the fracture site to open voltage-gated calcium channels and trigger the biological cascade leading to bone healing. The proposed mechanism of action is supported by clinical data suggesting that direct current electrical stimulation methods, where strong electric fields are applied directly to the fracture, appear to work better to promote bone healing than noninvasive stimulation methods^6^.

Furthermore, early data in vitro^11^ and in rodents suggest that noninvasive stimulation from the surface of the skin can work nearly or as effectively as invasive stimulation to promote bone growth, yet the positive results from these models have yet to be born out in clinically translatable large-animal models or clinical studies. One underappreciated factor that may explain this disconnect is the difference in scale between noninvasive stimulation in a rodent versus a human. Electric potential falls off from a bipolar electrode pair proportional to ~ 1/r^2^^7^, where r is the distance of the tissue from the electrode. In a rodent this distance is often 1 mm or less, whereas in a human the distance to the apex of the fracture can be 1 cm or more, meaning the same voltage applied externally may yield more than two orders of magnitude less electric potential at the site of fracture in humans than in rodents. However, there are no reports in the literature of measurements of electric field strength reaching the fracture site during noninvasive EBGS therapy. This is despite the criticality of the electric field strength at the fracture site to activating voltage-gated calcium channels—central to the proposed mechanism of action of EBGSs.

In this study, we—for the first time—use computational modeling and direct measurements from sheep cadaver metatarsus and tibia and human cadaver tibia cortex to create a framework for estimating the electric field strength reaching the fracture site during noninvasive EBGS therapy. We demonstrate that noninvasive stimulation at the skin in a large-animal model only generates a weak electric field at the deep bone fracture. We show that the electric field reaching the fracture site is highly dependent on the depth of the fracture and the electrode configuration. To address these limitations, we introduce a strategy using a novel electrode, the Injectrode, which can be simply injected into the bone fracture in a minimally invasive fashion, to increase the electric field strength reaching the deep fracture site during noninvasive EBGS therapy. Finally, we discuss the changing impedance of tissue at the fracture site during healing and its impact on the electric field reaching the fracture site during noninvasive EBGS therapy. This study lays the groundwork for a future in vivo large-animal sheep study planned by our group to test Injectrode-augmented EBGSs versus standard noninvasive EBGSs.

## Methods

First, we made direct measurements of the electric field strength reaching the fracture site in human tibia cadavers, and FEM computer models, to study the limitations of conventional noninvasive EBGSs. We then created another FEM model for a sheep leg in the metatarsus region based on dimensions from literature and measured in sheep cadavers and tissue conductivity values from literature (Supplementary Material 1). We used the sheep model to study optimal stimulation electrode configurations and the effect of healing stage on the electric field at the fracture site. Lastly, we modeled the Injectrode concept in both human and sheep and tested Injectrode prototypes in human tibia and sheep metatarsus and tibia cadavers.

### Cadaver measurements

A total of 5 adult human legs and 5 adult sheep legs were received frozen, kept at – 20 $^\circ$C, and thawed 48 h before the experiment. No experiments were performed on living humans or animals. The de-identified human cadaver legs were ethically obtained from the Anatomy Gifts Registry (Hanover, MD, USA) by donation. In accordance with the United States Federal Policy for the Protection of Human Subjects ('Common Rule'), research on the cadaver legs was not considered human subjects research and did not require institutional ethics approval. Human legs were excluded for known osteoporosis or bone cancers.

#### Surgical approach and fracture model

**Figure 1:**
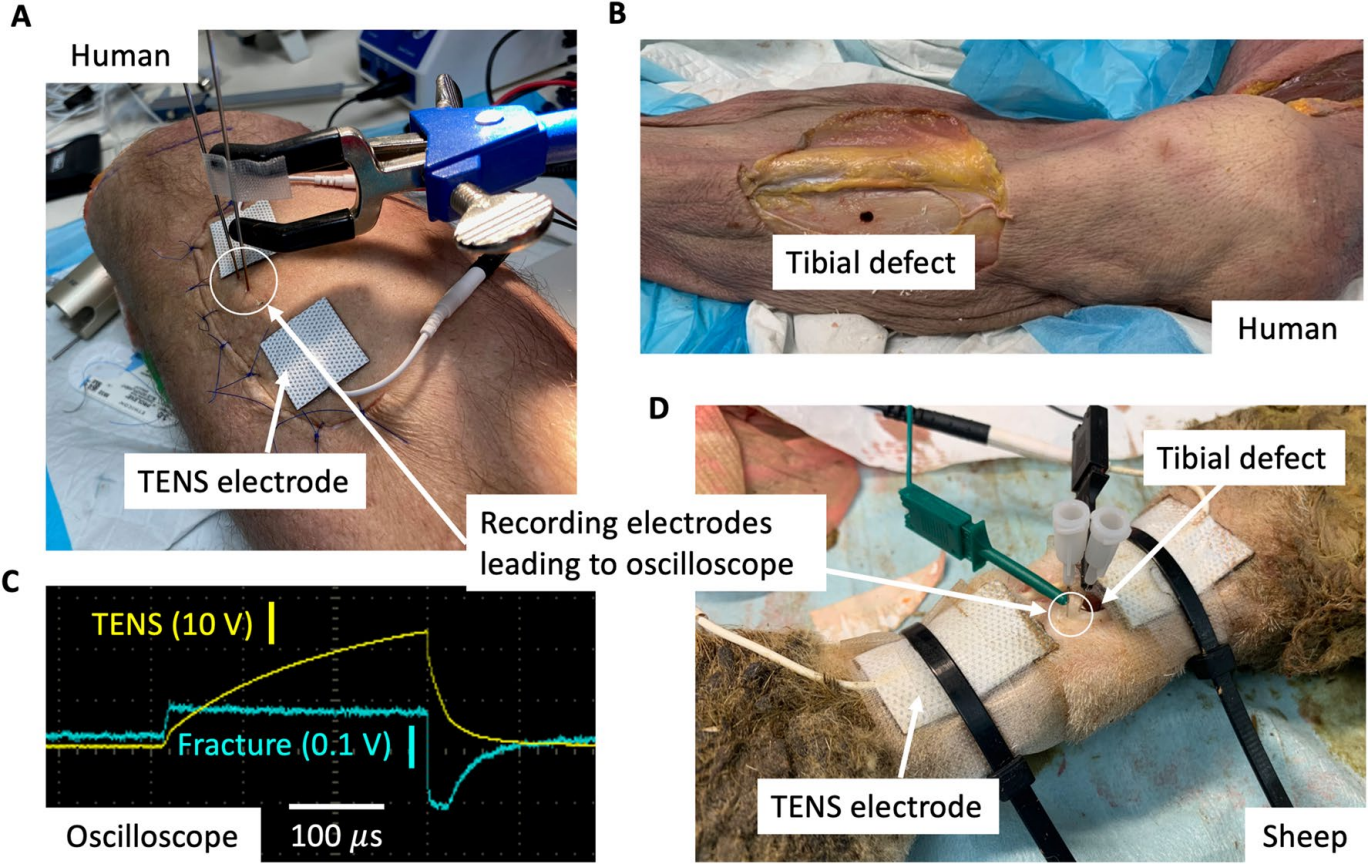
Direct electric field measurements at fracture site during noninvasive EBGS therapy. (**a**) Human cadaver leg instrumented with noninvasive stimulation electrodes akin to an EBGS. Surgical flap was created to access tibial bone to drill defect and two small holes for recording electrodes. Microneurography recording electrodes were inserted percutaneously into bipolar measurement holes in bone after the surgical flap was closed with sutures. (**b**) Tibial bone defect visible with surgical skin flap retracted. (**c**) Oscilloscope screenshot shows voltage delivered through EBGS TENS electrodes externally (yellow trace) and recording at tibial fracture defect internally (blue trace) in a human leg cadaver. (**d**) Recording setup similar to (**a**) in sheep leg cadaver with metatarsus fracture defect.

For human legs, the tibias were subdivided into three equal sections—proximal third, middle third, and distal third—with the tibial tuberosity as the most proximal landmark and the talocrural joint as the most distal landmark. After the limb was thoroughly shaved, within each section of the tibia, a crescenteric incision was made through the pretibial skin, subcutaneous fat, and periosteum (Fig. 1A,B). The flap of soft tissue was then reflected laterally en-bloc to expose the underlying anterolateral tibial cortex. We measured the pretibial fat thickness of the soft tissue flap for human cadaver limbs, which is the distance between the fracture defect and the stimulation electrodes. A crescenteric incision was selected to minimize interference with soft tissue planes directly under the stimulation electrodes. In this manner, three separate measurement sites were created per human cadaver leg—proximal third, middle third, and distal third. Two human legs were too short to be divided into thirds, so these were divided into halves. One sample was determined to be an outlier based on statistical anomality and a distinct raw waveform shape, suggesting poor contact between the recording electrode and bone (all raw waveforms shown in Supplementary Material 2). Thus, (n = 12) total measurements were available from human cadaver limbs. The sheep limbs (Fig. 1D) were not divided into thirds and one measurement was taken per limb (with the same approach and setup as described above for human limbs) for a total of (n = 5) measurements. Further, Nair™ hair remover was applied to the sheep leg for an extended duration as shaving was insufficient to remove the dense, thick wool coat of the sheep.

To create the fracture model, we drilled a unicortical 5.5-mm diameter defect^12,13^ in the center of the exposed cortex through the anteromedial tibia until cancellous bone was encountered. The depth of the defect was dependent upon cortical thickness of the tibia, which varied from subject to subject, and anatomical location along the longitudinal axis of the tibia. Drill holes (0.5-mm diameter) were made 2 mm and 5 mm from the defect along the longitudinal axis of the tibia for the recording electrode pair. The soft tissue flap was then closed in layers using 5–0 permanent suture to obliterate dead space. The recording electrodes were inserted percutaneously through the flap into the 0.5-mm diameter holes made previously in the cortical bone. The noninvasive stimulation electrode pair, separated by 2 cm, was placed across the 5.5 mm osseous defect on the skin overlying the defect.

#### Electrical stimulation

Electrical stimulation was delivered to the cadaver legs with a TDT electrical stimulator (Tucker-Davis Technologies, Alachua, FL, USA). Pulses at 5 mA with a pulse width of 300 $\upmu$s were administered at 25 Hz through two Transcutaneous Electrical Nerve Stimulation (TENS) electrodes (InTENSity, USA) cut into 1 × 1 inch squares. The two electrodes were positioned 2 cm apart on the skin overlying the defect. Delivery of the stimulation waveform was monitored on an oscilloscope through the duration of the experiment (Fig. 1C). The stimulation frequency and pulse width were selected to be representative of the wide range of parameters explored clinically and preclinically across EBGS modalities^3,14^. Further work is needed to identify optimal stimulation parameters.

#### Electric potential measurement

Measurements of electric potentials at the fracture were performed using a pair of shank-insulated microneurography electrodes, with exposed recording tips, inserted into the two 0.5-mm diameter holes, separated by 3 mm, adjacent to the osseous defect. An electrically conductive gel (Physio Control, Redmond, WA, USA) was placed inside the two 0.5-mm recording electrode holes before recording electrode placement to ensure good electrical contact of the recording electrode with bone. 3 mm was selected as the minimum distance we could reliably drill the second hole without concern of drill bit slippage and damage to the first hole. A smaller distance would give a better estimate of electric field at a point but could also introduce excessive electric field distortion, which occurs from the placement of the conductive measurement probe into less conductive tissue. We used an oscilloscope with differential probes to record the voltage (electric potential) waveform across the two recording electrodes inserted in the bone (Fig. 1C) to allow for good electrical isolation from ground.

Electric potential values between the measurement electrode pair were reported throughout this manuscript. To convert to electric field magnitude values (V/m), the electric potential (V) was divided by the distance between the measurement electrode pair (3 mm). Results were reported in electric potential to allow for more intuitive contextualization by EBGS therapy practitioners who typically program EBGSs in the units of ‘Volts’ or ‘mA’.

### Finite element method (FEM) model

**Figure 2:**
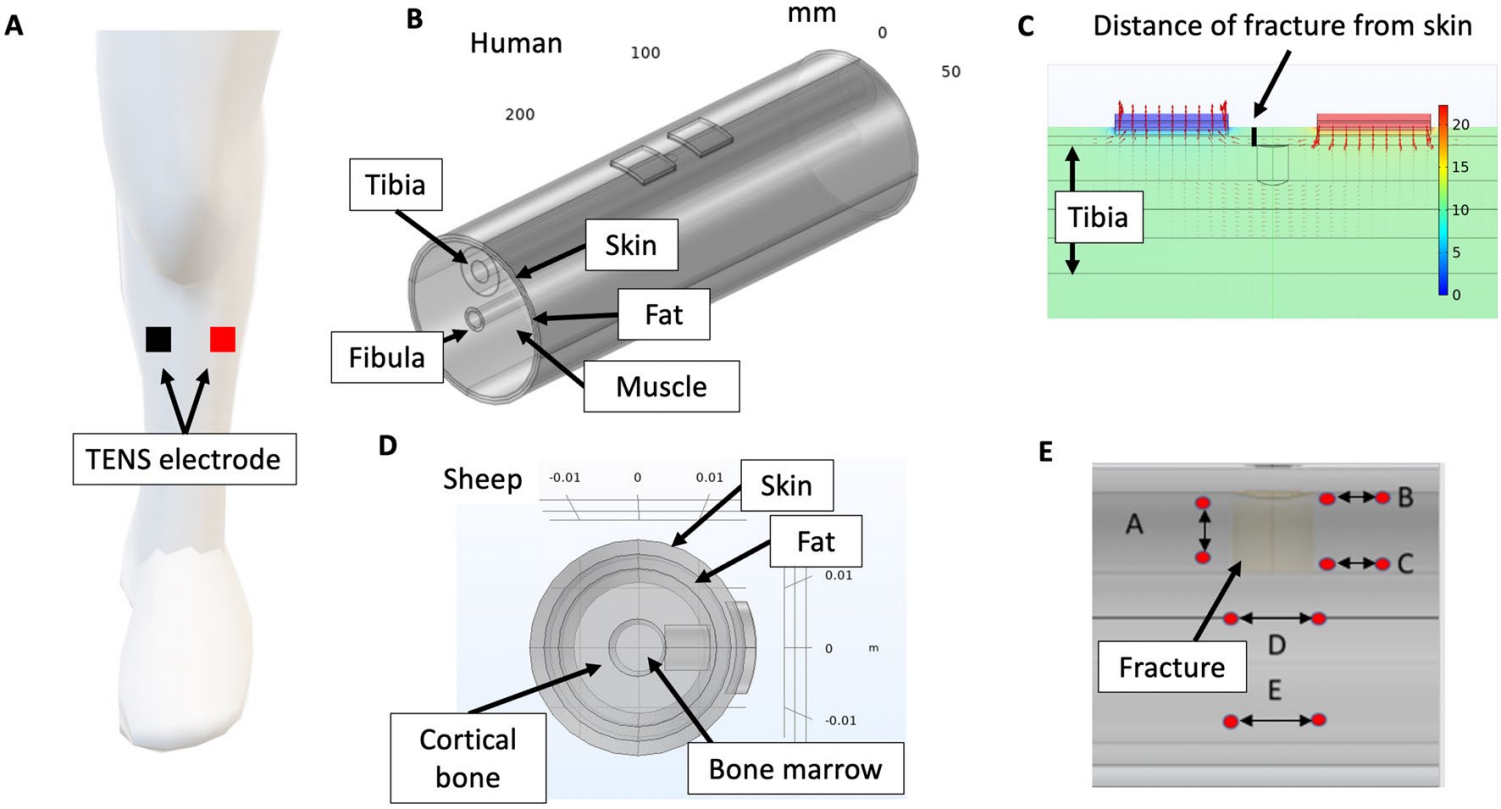
EBGS computational FEM modeling in humans and sheep. (**a**) EBGS electrodes deployed noninvasively (capacitive coupling) for tibial fracture. (**b**) 5-layer computational FEM model of human tibial defect with noninvasive EBGS electrodes patches as shown in (**a**). FEM model includes tibia and fibula bone. (**c**) Electric potential solution of FEM model in (**b**) showing rapid drop off in electric potential from stimulation electrodes to bone defect, even at a short distance without excess adipose tissue. (**d**) FEM model of sheep metatarsus defect with noninvasive EBGS electrodes patches. (**e**) Electric potential measurement points around bone fracture defect. Measurements of electric potentials were taken at five representative points (**A**–**E**) as the critical field direction required for bone healing has not been established in literature to date.

FEM models were set up in COMSOL Multiphysics (Burlington, MA, USA) representing simplified adult human legs (tibia region as shown in Fig. 2A) and sheep legs (metatarsus region), based on anatomical measurements, with a cortical defect in the anterolateral surface of the bone (Fig. 2B,D). The stimulation electrodes were modeled on the skin’s surface overlying the defect. For both humans and sheep, tissue conductivity values were derived from the literature or measured and are summarized in Supplementary Material 1.

#### FEM Model geometry

We built a 5-layer human lower leg model comprising of skin, fat, muscle, cortical bone, and bone marrow. Cortical bone thickness was set to 12.9 mm with external cortical bone diameter set to 28.8 mm^15^ in the human tibia model (Fig. 2B). For the sheep metatarsus model (Fig. 2D), based on an anatomical study in adult cross-bred ewes^16^, cortical bone thickness was set to 14 mm with external cortical bone diameter set to 22 mm. In both models, fat thickness and skin thickness measurements were not available in the literature. Instead, we measured these to be ~ 2 mm each (Fig. 2C). No muscle layer was included in the sheep model to reflect the anatomy of metatarsus. In both the human and sheep models, the bone defect was modeled as a cylindrical cutout of the cortical bone with a diameter of 5.5 mm with full thickness down to the medullary canal. The stimulating electrodes were modeled based on commercially available hydrogel TENS electrodes cut to 1 × 1 inch squares.

#### Stage of healing

We defined a high conductivity and a low conductivity material to fill the bone defect to represent early-stage and late-stage fracture healing, respectively^17^. The high conductivity group resulted from averaging the conductivities of dominant bone defect constituents during early bone healing—extracellular fluid, hematoma, and cartilage (Supplementary Material 1). The low conductivity group resulted from averaging collagen and cortical bone conductivities (Supplementary Material 1). These computations generated values of 1.18 S/m and 0.005 S/m, respectively.

#### FEM solution specifications

The COMSOL stationary study was used to calculate the electric field at the fracture site during noninvasive electrical stimulation. The COMSOL stationary study is a direct current (DC) analysis of the steady-state electric potentials in the modeled volume when a constant electrical stimulation is delivered. A ‘fine’ mesh was used in both models with 312,438 tetrahedral elements in the human model, and 67,100 tetrahedral elements in the sheep model. Use of the ‘fine’ mesh setting was determined through a mesh convergence analysis^18^. For the monopolar configuration of the surface stimulation electrodes, all sides of the model were set as ground to mimic a distant return electrode. For bipolar configurations of the surface electrodes, a current-controlled stimulation was delivered between the two electrodes using a Dirichlet boundary condition^19^. All FEM model simulations were solved with electrical stimulation delivered at 1 mA. Electric potential results were scaled by a factor of five to match the cadaver-delivered stimulation current of 5 mA.

Ohm’s law in vector form was used to calculate the spread of current from the surface stimulation electrodes and the corresponding electrical potentials (voltages) throughout the modeled volume. Electric potential at the drill hole fracture site was reported between five paired points (3 mm pair length) around the fracture hole in the model (Fig. 2E) because the critical direction relevant to bone healing is currently unknown. The five measurement pairs were selected to provide a representation of the electric field strength at and around the defect in the longitudinal and transverse directions.

#### Stimulation electrode configuration exploration in silico

**Figure 3:**
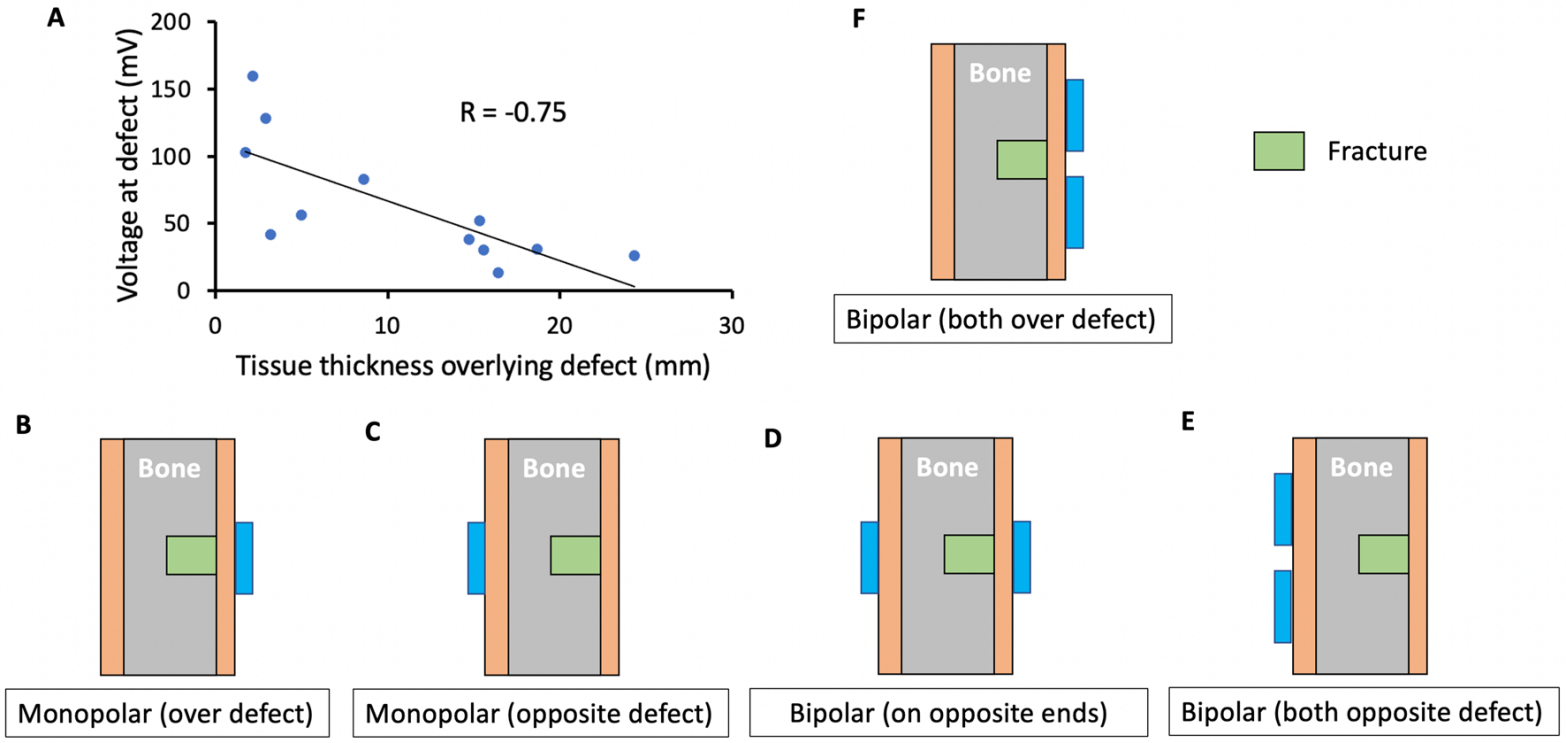
Results of noninvasive EBGS FEM models in sheep investigating stimulation electrode configurations. (**a**) Increased distance between the fracture defect and the stimulation electrodes is correlated to decreased voltage at the defect site during noninvasive EBGS therapy. This supports our hypothesis that EBGSs are critically limited in humans and large-animal models due to the weak electric fields reaching the fracture site. (**b**) Electric potential solution with stimulation electrode over defect site in monopolar configuration with distant return. (**c**) Electric potential solution with stimulation electrode opposite defect site in monopolar configuration with distant return. (**d**) Electric potential solution with stimulation electrodes in bipolar configuration: one over defect site and one opposite defect site. (**e**) Electric potential solution with stimulation electrodes in bipolar configuration: both opposite defect site. (**f**) Electric potential solution with stimulation electrodes in bipolar configuration: both over defect site. This configuration was used in all reported cadaver measurements.

Several combinations of different monopolar and bipolar stimulation electrode configurations were tested in the FEM model (Fig. 3B–F). Each configuration’s resulting electric potential at the defect was measured at five different pairs of points in the model, shown in Fig. 2E. These paired points represented the magnitude of the electric field generated by the surface stimulation electrodes at the fracture site.

In the cadaver work, we used a bipolar stimulation electrode configuration with both electrodes over the defect (Fig. 3F) and recording electrode point B. Both these choices were motivated by computational modeling results and practical limitations on the set-up. Practically, there were limitations on where the recording electrode could be inserted with minimal disruption to the anatomy and where the stimulation electrodes could be placed without creating a confound of a direct conductive path through skin by way of the edges of the surgical skin flap.

### Injectrode concept

**Figure 4:**
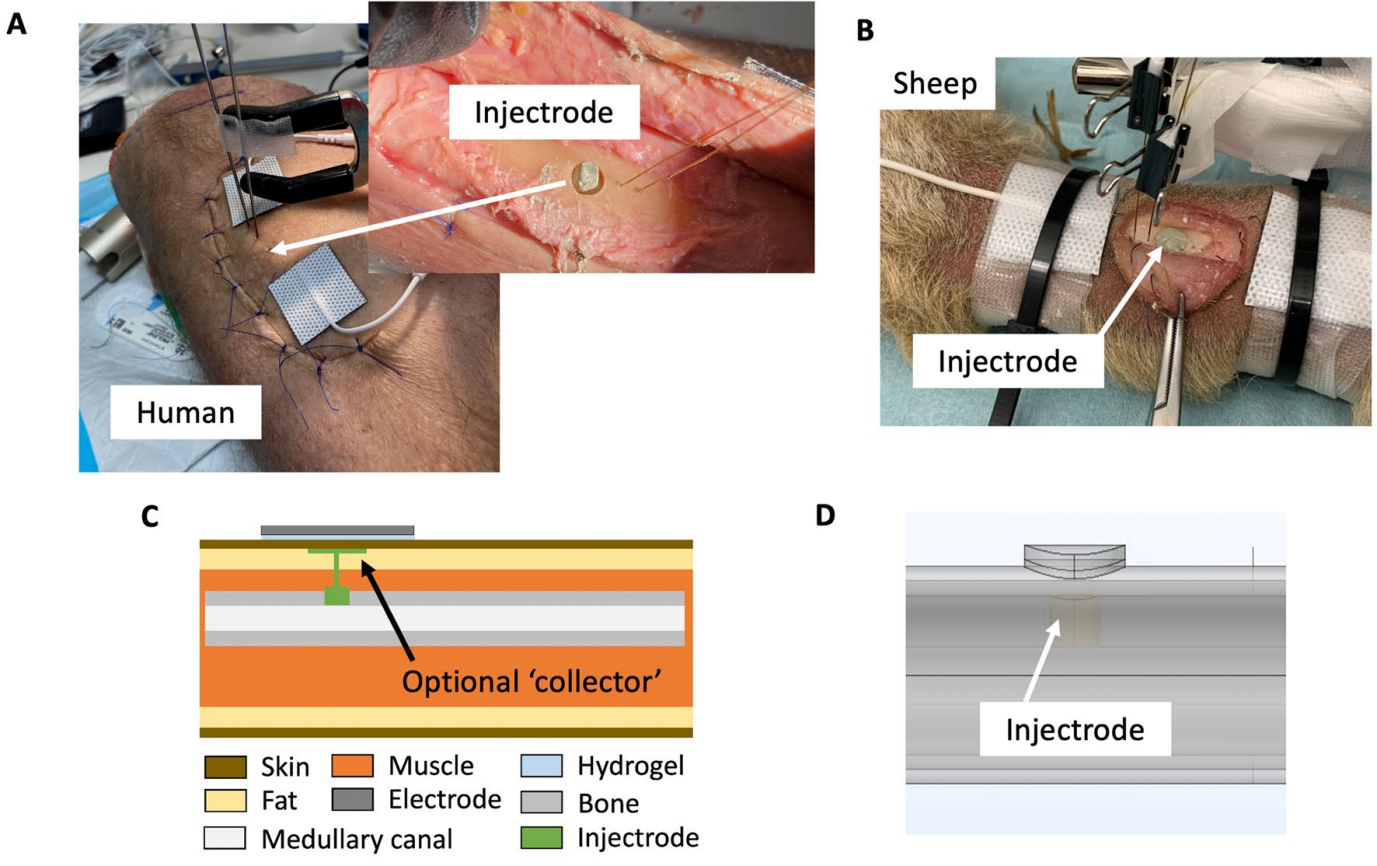
Injectrode to augment electric field reaching the fracture site during noninvasive EBGS therapy. (**a**) Addition of Injectrode to fracture defect under surgical flap in human cadaver. (**b**) Addition of Injectrode to fracture defect in sheep cadaver. (**c**) Illustration of Injectrode ‘collector’ placed subcutaneously with injected Injectrode wire leading to more Injectrode at the fracture site (monopolar configuration). Thereby, routing current from the surface of the body to the deep bone and increasing electric field strength reaching the fracture site. Note that the Injectrode ‘collector’ is optional for superficial fractures but will be beneficial for deep fractures. (**d**) Injectrode in the FEM model. 3D representation of schematic in (**c**) without optional Injectrode ‘collector’.

We utilized an in-body curing injectable electrode, the Injectrode, to address the goal of increasing the electric field strength at the fracture site to promote calcium channel activation^8^ and aid bone healing. Injectrode begins as an uncured, free-flowing prepolymer^20^. This prepolymer sets into a solid in the body and forms a conductive interface with the target tissue. In principle, the benefit of this minimally invasive electrode is that it offers increased stimulation strength by targeting the fracture site directly, similar to direct current electrical stimulation (DCES), by routing current from the EBGS TENS electrodes to the fracture site^18^. An Injectrode ‘collector’ can be added subcutaneously and routed to the bone defect in cases of a deeper fracture (Fig. 4C). The Injectrode is a minimally invasive method for delivering electrical stimulation that can be injected sterilely under imaging guidance and remains entirely subcutaneous. Energy is wirelessly delivered to the Injectrode using transcutaneous coupling from external surface electrodes^18^. In this manner, there is no open wound, and the technique is hypothesized to be less susceptible to infections while increasing the electric current reaching the fracture site.

A silver-particle-based Injectrode prototype, formulated for preclinical studies, was used in this study to demonstrate proof of concept. Injectrode material used in this study was manufactured by Neuronoff, Inc. (Cleveland, OH, USA) using a polymer-conductor variant of the Injectrode as described previously^20^. Two parts of Pt-curing silicone elastomers (World Precision Instruments, FL, USA) with metallic silver particles (Sigma-Aldrich, MO, USA) were mixed and loaded into a 5 mL syringe.

After data collection was performed without the Injectrode, the sutures were released, and the flap was re-elevated. The Injectrode was administered in its elastomer state into the 5.5 mm osseous defect until the surface of the elastomer was flush with the surrounding cortical bone (Fig. 4A,B). The flap was then closed, and the recording and stimulation electrodes were placed. Data collection was repeated as it was performed without the Injectrode material in place. This way, each cadaver limb acted as its own control (within-subject design), and the electric potentials measured with the recording electrodes were compared in the non-Injectrode versus the Injectrode configurations to calculate a ‘gain’ value.

We directly compared measurements of the electric potential at the fracture site between the Injectrode and non-Injectrode states measured in cadavers to the values predicted by the computational FEM models. The Injectrode material was modeled to completely fill the bone defect and have the conductivity of a conductive metal (4e7 S/m) (Fig. 4D).

### Statistical analysis

One-sample one-tail t-tests were used to test the null hypothesis (H_0_: gain = 1) against the alternate hypothesis (H_1_: gain > 1) for human and sheep cadavers. One-sample t-tests were selected over paired t-tests as the hypothesis was that Injectrode would augment the electric potential reaching the fracture. The one-sample one-tail t-test offered the simplest method to test this hypothesis, while the calculation of the gain value (ratio of electric potential at fracture without Injectrode to electric potential with Injectrode) further collapsed the variability in electric potential values between cadaver legs. One-sample t-tests and correlation analysis were run in Microsoft Excel 2018. The Shapiro–Wilk test was used to verify the normality assumption of the student t-tests. Type II error tolerance was set at 5% ($\alpha =0.05$). The study was not pre-registered, and findings should be considered exploratory and not confirmatory.

## Results

In the following results section, we first report on measurements of the electric field strength at the fracture site of a human cadaver leg during noninvasive EBGS therapy. These measurements support our hypothesis that the efficacy of noninvasive EBGS therapy may be critically limited by the electric field reaching the fracture site. We then present a computational FEM model of the human leg to study the effect of stage of healing on the electric field strength reaching the fracture site during EBGS therapy. Subsequently, we created a model for sheep, a preclinical large-animal model for fractures. We used the sheep FEM model to explore stimulation electrode configurations and electric field measurement points around the fracture site.

Observing the limited electric field strengths reaching voltage-gated calcium channels within the bone during EBGS therapy, we evaluated the Injectrode as a minimally invasive method to augment these weak electric fields. We provide modeling results and cadaver measurements in sheep and humans to support the use of the Injectrode in an upcoming in vivo preclinical study. Lastly, we present results on the effect of stage of healing and fracture depth on electric field reaching the fracture site during Injectrode-augmented EBGS therapy.

### First measurements of electric field at the fracture site during EBGS therapy

In a rodent the distance between the fracture site and the noninvasive EBGS electrodes is often 1 mm or less, whereas in a human the distance to the apex of the fracture can be 1 cm or more (mean of 1.1 cm in this study). The electric potential falls off from a bipolar electrode proportional to ~ 1/r^2^^7^, where r is the distance between the electrode pair and the bone. This suggests that the same voltage applied externally may yield more than two orders of magnitude less electric potential at the fracture site in humans than in rodents. We hypothesize that the weak electric fields reaching the fracture site in humans explains the loss of efficacy in EBGS therapy from preclinical rodent testing to human clinical results.

To investigate our hypothesis, we made direct measurements of the electric potential at the fracture site during EBGS therapy in human cadavers. During 5 mA of externally applied stimulation through 1 × 1 inch square TENS electrodes, generating a voltage of ~ 15–30 V, only 64 mV (45 mV standard deviation (SD)) reached the fracture site on average (Table 1). Figure 1C shows representative traces of the voltage at the external TENS electrodes and internal fracture site during measurements in a human cadaver tibia. Converting electric potential values to electric field strength, only 21 V/m reached the fracture site when ~ 1100 V/m was applied externally.

**Table 1:** Summary of electric potentials reaching fracture site in humans.

Human	Measurement pair B (mV)
Model: Early-stage healing	29.7
Model: Late-stage healing	22.0
Cadaver (n = 12)	64 SD 45

The small ratio, ~ 1.9%, of the externally applied electric field to the electric field at the fracture site in humans highlights why EBGS therapy may be critically limited in its clinical efficacy, while still performing well in small animal models, where there is a shorter distance between the noninvasive stimulation electrodes and the fracture site. In sheep cadavers, where the distance from the surface of the skin to the apex of the fracture is ~ 2–5 mm (intermediate to rodents and humans), we measured an average voltage of 680 mV (SD 550 mV) at the fracture site (Table 2). Further supporting our hypothesis that the distance between the fracture site and external stimulation electrodes is critical, we observed that with increased pretibial fat thickness in humans (distance between the fracture site and surface EBGS electrodes), the voltage measured at the fracture site decreased (R = − 0.75), shown in Fig. 3A.

**Table 2:** Summary of electric potential at fracture site in sheep.

Sheep model: early-stage healing	Measurement pair (mV)				
Stimulation electrode configuration	A	B	C	D	E
Monopolar (over defect)	751	1773	2677	0	0
Monopolar (opposite defect)	54	152	34	0	1
Bipolar (on opposite ends)	940	1305	2430	0	0
Bipolar (both opposite defect)	35	475	555	435	1235
Bipolar (both over defect)	840	5385	3725	1295	1125
Cadaver: bipolar (both over defect) (n = 5)	NA	680 SD 550	NA	NA	NA

### Computational model of noninvasive EBGS therapy in humans

We constructed a computational FEM model of a human leg with representation of skin, muscle, fat, and the tibia and fibula bone. The computational model calculated 29.7 mV (early-stage healing) and 22.0 mV (late-stage healing) reaching the fracture site during 5 mA of stimulation delivered noninvasively (Table 1). The cadaver measurements, expected to be reflective of a stage of healing between early- and late-stage due to tissue preparation, resulted in a voltage of 64 mV (SD 45 mV) at the fracture site. The model prediction was within 1 SD of the cadaver measurements, which is an expected deviation considering the known variations in tissue conductivity at low frequencies^18^. The model underpredicted the average measured voltage at the fracture site by 54–66%. The error may be coming from the difference in skin impedance, a sensitive parameter, between the model and the cadaver. The cadaver skin impedance was likely higher due to the dehydration of the tissue and therefore required a greater voltage to deliver 5 mA of noninvasive stimulation. We calculated the voltage across the TENS electrode in the FEM model to be 3.8 V at 5 mA of stimulation compared to the 15–30 V in the cadavers (See Supplementary Material 2). The higher voltage across the TENS electrodes in the cadaver would result in a correspondingly higher voltage at the fracture site, compared to the FEM model.

We computed the ratio of electric field applied externally to the electric field at the fracture site to compare to the average 1.9% ratio measured in the human leg cadaver. The human FEM model predicted a ratio of 1.9% for late-stage healing and 2.6% for early-stage healing compared to the 1.9% measured in the cadaver. This improved agreement of electric field ratios between model and cadaver suggested that the model captured the spread of electric field in tissue accurately, even when the absolute electric field values were inaccurate. Therefore, trends in the FEM model may be accurate even when absolute values were not.

### Stage of healing and electric field at fracture site with conventional EBGSs

The evolving biological process at the fracture site^17^ during healing leads to different tissue electrical conductivities at the fracture site over the course of the healing process. In general, the conductivity at the fracture site decreases during healing, starting with a conductivity similar to extracellular fluid or hematoma and ending with the conductivity of cortical bone. This influences the electric field reaching the fracture site during use of an EBGS. More electric current reaches the fracture site in the acute stage of healing as the fracture is filled with higher conductivity material and represents a preferential, lower resistance, path for current to travel. For this reason, we computed model values for both early-stage and late-stage healing (Table 1).

### Electric field at fracture site during EBGS therapy in sheep

We used a computational FEM model of a sheep leg to explore several different stimulation electrode configurations and identify optimal configurations for an upcoming sheep EBGS study planned by our group. We also used the model to explore electric field measurements at different points around the fracture site (Fig. 2E) because the critical direction relevant to bone healing is currently unknown.

We created a FEM model for a sheep leg in the metatarsus region based on dimensions from literature and measured in sheep cadavers. The model calculated 5385 mV reached the fracture site during 5 mA of noninvasive stimulation, while sheep cadaver (n = 5) measurements averaged 680 mV (SD 550 mV) at the fracture site (Table 2). The model overpredicted the average measured voltage at the cadaver fracture site by 690%. The error may again be due to the difference in skin impedance between the model and the cadaver, a sensitive parameter^18^. A sensitivity analysis on the sheep metatarsus FEM model showed that skin conductivity was a key parameter that affected the ratio of electric field at the fracture site to the externally applied electric field (Supplementary Material 3). The cadaver skin impedance could be substantially lower due to the application of Nair™ for an extended duration to remove the thick hair of the sheep. The Nair™ could have damaged the high impedance outer layer of skin (stratum corneum in the epidermis). With a lowered skin impedance, we would have required a lesser voltage to deliver 5 mA of noninvasive stimulation into the skin. The lesser voltage across the TENS electrodes in the cadaver would result in a lesser voltage at the fracture site compared to the FEM model. To test this hypothesis, we adjusted the skin layer conductivity in the model from 8.0e−4 S/m (dry skin^21^) to 4.34e-1 S/m (dermis^22^) to model skin with the outermost epidermis layer compromised by the extended application of Nair™. The updated FEM model calculated 536 mV reached the fracture site during 5 mA of noninvasive stimulation—comparable with the cadaver measurements of 680 mV (SD 550 mV). However, for the remaining study, we used the original conductance of skin (8.0e−4 S/m) in the sheep model. We did this to retain the relevance of the sheep model findings to the planned in vivo Injectrode-augmented EBGS sheep study, where the skin preparation will be more conservative and should not damage the epidermis.

Several electrode configurations were explored in the FEM model to identify suitable configurations for an upcoming sheep EBGS study planned by our group. Stimulation electrode configuration (Fig. 3B–F) and measurement point (Fig. 2E) had a profound impact on the electric field value. The stimulation electrode configuration ‘bipolar (both over defect)’ produced the highest electric field at the fracture site at measurement point B (5385 mV). This motivated use of point B and stimulation electrode configuration ‘bipolar (both over defect)’ to maximize the signal to noise ratio for cadaver electric potential measurements.

### Computational models and cadaver experiments support use of Injectrode to augment EBGS therapy

The Injectrode, a minimally invasive conductive electrode, may be injected under image guidance to the fracture site to guide current delivered noninvasively towards the fracture site. We hypothesize that utilizing Injectrode at the fracture site will augment the electric field reaching the bone during noninvasive EBGS therapy. To investigate the efficacy of the Injectrode to augment the electric field reaching the fracture site, we modified the previous sheep and human computation models to include the Injectrode. We also made measurements in the same cadaver models with the addition of Injectrode in the fracture. We report on the ‘gain’ in electric field at the fracture site (point B in Fig. 2E) with Injectrode compared to without Injectrode. A value greater than one indicates efficacy of the Injectrode.

Computational modeling for the human tibia in the late-stage healing state (low conductivity tissue at fracture site) resulted in a 37% gain in electric field at the fracture site with the addition of the Injectrode (Table 3). For the early-stage healing state (high conductivity tissue at fracture site), the gain was a lesser 2% with the addition of the Injectrode. Human cadaver measurements (n = 12), which are likely reflective of a state between early- and late-stage healing, showed an average gain of 23% (SD 22%, p = 0.002, H_0_: gain = 1, H_1_: gain > 1, one-tail). There was an error of 11–17% between FEM model early- and late-stage healing and human cadaver measurements. Note that we consider these findings exploratory and not confirmatory because the measurement and statistical analysis methods were not pre-registered but determined post-hoc instead.

**Table 3:** Human results of Injectrode-augmented EBGS.

Human	Measurement pair B (gain)
Model: early-stage healing	1.02
Model: late-stage healing	1.37
Cadaver (n = 12)	1.23 SD 0.22

**Figure 5:**
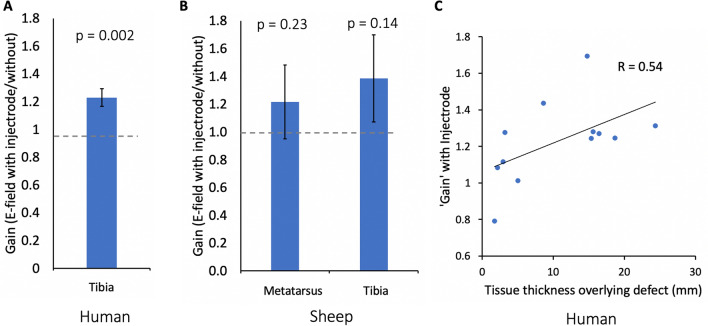
Injectrode increases electric field at fracture site when used with noninvasive EBGS therapy. (**a**) ‘Gain’ in electric field at tibial fracture defect with Injectrode-augmented EBGS therapy in human leg cadavers. Error bars indicate standard error of the mean (SEM). (**b**) ‘Gain’ in electric field at independent tibial and metatarsus fracture defects with Injectrode-augmented EBGS therapy in sheep leg cadavers. (**c**) Scatter plot showing increased utility of Injectrode to augment electric field at fracture site at increased bone depths.

FEM modeling for the sheep metatarsus predicted a 69% gain in electric field at the fracture site with the addition of the Injectrode in the late-stage healing state (low conductivity tissue at fracture site) (Table 4). In the early-stage healing state (high conductivity tissue at fracture site due to initial edema buildup), there was a lesser 1% gain predicted by the model with the addition of the Injectrode to the metatarsus fracture. Cadaver measurements at the superficial metatarsus bone (mimicking the model) showed a gain of 22% (SD 59%, p = 0.23, H_0_: gain = 1, H_1_: gain > 1, one-tail), shown in Fig. 5A. The sheep metatarsus cadaver measurements aligned with the FEM model predictions with an error of 17–39% between model early- and late-stage healing and the cadaver setup—despite larger errors in the absolute voltage values at the fracture site (as discussed earlier in this paper). The sheep FEM model is therefore still useful for studying relative trends including the ‘gain’ ratio between the electric field at the fracture site with and without Injectrode. The sheep cadaver ‘gain’ increase was not statistically significant, in large part due to the higher variability (standard deviation) in the data. The methods for recording voltages at the bone defect in cadavers were initially developed during these sheep cadaver experiments, before the human cadaver measurements, and could explain the larger standard deviation of the sheep cadaver measurements compared to the human cadaver measurements.

**Table 4:** Sheep results of Injectrode-augmented EBGS.

Sheep metatarsus model: early-stage healing (unless specified)	Measurement pair (gain)				
Stimulation electrode configuration	A	B	C	D	E
Monopolar (over defect)	0.94	1.00	1.02	NA	NA
Monopolar (opposite defect)	0.94	1.02	0.85	NA	NA
Bipolar (two electrodes on opposite end)	0.94	0.99	1.02	NA	NA
Bipolar (both opposite defect)	1.00	1.01	1.02	0.99	1.00
Bipolar (both over defect)	0.97	1.01	1.01	− 0.98	− 1.00
Bipolar (both over defect), late-stage healing	0.59	1.69	1.66	− 0.65	− 0.88
Cadaver: bipolar (both over defect) (n = 5)	NA	1.22 SD 0.59	NA	NA	NA

### Depth of fracture increased utility of Injectrode-augmented EBGS therapy

We found that the Injectrode provided more benefit over conventional EBGSs at greater depths of the fractured bone from the surface of the skin. With increased pretibial fat thickness in humans (distance between fracture site and surface EBGS electrodes), the ‘gain’ in electric field at the fracture site with the Injectrode compared to without increased (R = 0.54), shown in Fig. 5C. Furthermore, cadaver measurements at the deeper sheep tibia showed a gain of 39% (SD 70%, p = 0.14, H_0_: gain = 1, H_1_: gain > 1, one-tail) compared with the superficial metatarsus bone, which showed a gain of only 22% (SD 59%, p = 0.23, H_0_: gain = 1, H_1_: gain > 1, one-tail), shown in Fig. 5B.

### Stage of healing and electric field at fracture site with Injectrode-augmented EBGS

Data in Tables 3 and 4 showed that, as with conventional noninvasive EBGSs, electric potential at the fracture site during Injectrode-augmented EBGS therapy was also affected by the tissue electrical conductivity in the fracture site as it changed with the stage of healing. Opposite to conventional EBGSs, Injectrode was most advantageous after early stages of healing instead of during. This is because conductive edema acts, to some extent, similarly to the Injectrode, especially for more superficial defects. Computational modeling with the Injectrode showed a 34% and 67% greater electric field, in humans and sheep respectively, at the fracture site during later stages of healing compared to earlier stages of healing.

## Discussion

In this study, we reported on electric fields measured at the fracture site during noninvasive EBGS therapy. Strength of the electric field at the fracture site is believed to be critical to the proposed mechanism of action of EBGSs—the activation of voltage-gated calcium ion channels^8^. We investigated the electric field strengths reaching the fracture site during noninvasive EBGS therapy in sheep and human cadavers and computational models. We observed minute electric fields reaching the fracture site. On average, only ~ 1.9% of the externally applied electric field reached the fracture site in human cadavers. We reported on the effects of fracture depth and healing stage on the electric field strength reaching the fracture site. We then proposed and investigated the use of a minimally invasive injectable electrode, the Injectrode, to augment the electric field reaching the deep fracture site. The computational and cadaver models in this study lay the groundwork for a preclinical sheep study planned by our group to investigate the use of the Injectrode in vivo to augment electric field strength reaching the fracture site—and consequently improving time to fracture healing—during EBGS therapy.

To our knowledge, this is the first study to characterize electric field strengths at the fracture site during noninvasive EBGS therapy. This marks a critical step towards building consistent efficacy of EBGSs in human patients. We measured only ~ 1.9% of the externally applied electric field reaching the fracture site in human cadavers. This translates to an electric field strength of 21 V/m at the fracture site during 5 mA of noninvasive stimulation. The 21 V/m electric field strength is low compared to neural activation thresholds, which are reported to be > 100 V/m^23^. Neural activation is predicated on voltage-gated sodium channel activation while bone healing is putatively based on voltage-gated calcium channel activation, so differences in activation threshold are expected between sodium and calcium channels. However, the known neural activation thresholds contextualize the weak electric field strengths reaching the fracture site during noninvasive EBGS therapy in humans—even on the most superficial of bones. There are notable parallels between bone stimulation and neurostimulation and both fields may benefit from adopting techniques used in the other^11^.

Though there are many promising preclinical studies examining the effects of electrical stimulation for a wide array of osseous insults, many use custom stimulators and provide incomplete device specifications, making it difficult to repeat the experiment or translate the findings^3^. Moreover, many of these studies do not measure the electric field strength reaching the fracture site^3^. Directly measuring electric field in the cortex of the bone is critical to understand the field strengths required for a therapeutic effect and is also critical to scale from culture and small animal studies to large animal and humans^24^. The authors recommend future studies in the bone stimulation field employ direct measurement strategies.

Both computational and cadaver models in our study showed that (1) conventional EBGSs deliver lower electric fields to deeper fracture sites (Fig. 3A) and that (2) Injectrode-augmented EBGSs provides more of a benefit (‘gain’) over conventional EBGSs at deeper fracture sites. These findings explain why, in a previous study on the Injectrode for noninvasive neural stimulation^18^, the Injectrode was shown to improve neural activation by more than an order of magnitude over conventional noninvasive stimulation, while in the current study, the Injectrode is showing a more limited benefit of a 37% increase in electric field at the fracture site. The current work is studying the tibial bone, where the fracture is at a depth of only a few mm from the stimulation electrodes, while in the previous neural study, the nerve of interest was at a depth of ~ 2 cm from the stimulation electrodes. Overall, this makes deeper bones, such as the femur, more ideal to show the benefit of Injectrode-augmented EBGSs over conventional EBGSs. For fractures at the depth of the femur, the optional Injectrode ‘collector’ (Fig. 4C) may be required to realize the full potential of the Injectrode.

The two findings highlighted in the previous paragraph also explain why the Injectrode was shown to have a statistically significant benefit in augmenting electric field at the fracture site in human but not sheep cadavers. The depth of the fracture was deeper on average in humans, due to pretibial fat, compared to sheep. This resulted in weaker electric fields at the fracture site with conventional EBGS therapy and more of a ‘gain’ with the Injectrode. The larger effect size contributes greatly to establishing statistical significance at small sample sizes.

Our findings also characterize the effect of the stage of bone healing on efficacy of conventional noninvasive EBGSs. With a conventional EBGS, the electric field reaching the fracture is higher during the acute stages of healing, when high conductivity tissue fills the fracture—acting to route current to the deep fracture site. However, with an Injectrode-augmented EBGS, the ‘gain’ in electric field at the fracture is higher during the later stages of healing—when low conductivity tissue fills the fracture. The higher ‘gain’ of the Injectrode at later stages of healing has positive implications towards the delivery of Injectrode after a fracture. The Injectrode does not need to be administered immediately in the acute stages of healing, but rather can be administered even in the later stages of healing—widening the therapeutic time window available for its application.

Inductive coupling is another noninvasive EBGS modality that exists in the form of pulsed electromagnetic field (PEMF) therapy, which uses pulsed currents through solenoids arranged parallel to the skin to generate an alternating magnetic field. The alternating magnetic field induces an alternating electric field in tissue. PEMF therapy suffers reliability issues with electric field strength variation due to variation in the placement of the device and the thickness of enveloping soft tissue layers^24^. PEMF devices would benefit from a similar study as reported in this paper with direct measures of the electric field reaching the bone.

There were discrepancies between the computational model and cadaver measurements in absolute electric field value, especially in the sheep model. This was explained by the differences in tissue conductivity—especially the skin layer, after freezing and thawing in the cadaver and the application of Nair™ for hair removal—compared to tissue conductivity values from literature performed in living subjects or fresh tissue samples used in the FEM model. Although these differences in tissue conductivity make absolute electric field results from the model less predictive, relative results and trends still hold. An example of a relative result is the ‘gain’ value calculated for the model, which was the ratio of electric field at the fracture site with Injectrode compared to without. A better agreement was seen between cadaver and computational models in these gain values for both the human and sheep model.

Also due to differences in tissue conductivities, the cadaver measurements are likely reflective of values between early-stage and late-stage healing. The cadavers were frozen and then thawed, which created accumulation of high conductivity fluid in the leg. At the same time, due to the lengthy duration since the death of the subject, some tissue had congealed, forming lower conductivity spaces. Overall, the cadaver is likely more reflective of early-stage healing due to the accumulated fluid in the fracture drill hole from thawing of the cadaver limbs. For these reasons, we compared cadaver measurements to both early-stage and late-stage results from the FEM model. Opening and then suturing of the soft tissue flap in the cadaver legs could also have changed the conductivity of the thin fascial tissue layers compared to the intact state. Performing a similar protocol in a live anesthetized animal, or in a chronic experiment where scar formation occurs, would provide a more accurate picture of the electric fields reaching the fracture site during noninvasive EBGS therapy.

Further limitations of the experimental and modeling set-up may also explain the differences between the computational model and cadaver results. Firstly, the computational model assumed uniform thickness of fat and skin across the tibia. The human cadaver model in comparison is not a uniform cylinder and was subjected to wider variations. Secondly, the depth of the defect from the stimulation electrode has a profound effect on the electric potential reaching the fracture (~ 1/r^2^ relationship, where ‘r’ is distance)^7^. To this end, in the FEM model, we had a single distance between the defect and the stimulation electrodes, representing the average distance in cadavers. In each cadaver, this distance varied and may have contributed greatly to the subject-to-subject variation observed in the cadaver results.

The unicortical fracture model we used in this study is established to study bone healing and novel therapeutics^12,13^. While it does not fully capture the clinically relevant nonunion application of EBGS, for which an osteotomy ‘gap’ model may be more relevant, it does provide the capacity for high throughput testing with reduced risk to the animal. The unicortical fracture model was ultimately selected here to maximize the relevance of this study to the future chronic in vivo sheep study planned by our group to study Injectrode-augmented EBGSs versus standard noninvasive EBGSs.

The ideal EBGS would be relatively low cost and easy to administer, produce reliable stimulation, regardless of patient position or body habitus, and pose a minimal infection risk. Direct current electrical stimulation (DCES), as previously described, poses a significant infection risk and requires surgeries to implant and remove the device. Noninvasive stimulation (capacitive coupling) is an attractive option as it is noninvasive, thereby minimizing infection risk, and easy to use. However, the penetration of the electrical field into tissue is proportional to ~ 1/r^2^ with ‘r’ being the distance from the electrode^7^, making it useful only for superficial fractures. Finally, PEMF is noninvasive, and the magnetic field penetrates tissue well, but the devices are expensive and the electric fields at the fracture site are weak and heavily contingent upon device positioning and the thickness of tissue layers specific to each patient^24^. The sensitivity to position also makes PEMF heavily operator dependent and potentially inconsistent from application to application.

The Injectrode has the potential to provide reliable stimulation strength similar to DCES without the need of open surgeries for implantation and removal, which lead to increased risk of infection. To this end, the Injectrode has already been validated to produce neural effects at similar levels as conventional electrodes for dorsal root ganglion stimulation in a cat model^25^. While there are significant differences between the conductivity of bone and nerve tissue, Injectrode-augmented noninvasive EBGS therapy may offer a promising solution for the treatment of osseous nonunion and acute fractures prone to nonunion.

The Injectrode formulation used in this study was an early silver-particle-based prototype—only for use in cadaver and acute preclinical studies due to the toxicity of silver. It was used in this study to demonstrate proof of concept of electric field increase at the fracture site with Injectrode-augmented noninvasive EBGS. Other electrically conductive materials would have a similar effect of increasing electric field at the fracture site. Future versions of the Injectrode under development are based on gold or platinum microwires. Future work will need to investigate biocompatibility of the Injectrode, degradation kinetics, and its suitability to be injected at the fracture site. Past work includes testing the biocompatibility of the Injectrode in humans^26^.

This computational study lays the groundwork for a future preclinical study planned by our group to investigate augmenting conventional EBGSs with Injectrode to increase the electric field strength reaching the fracture site. While the Injectrode prototypes used in this study were used for proof-of-concept testing, newer generations of the microwire-based devices have higher translational potential. In the later stages of healing, we showed in sheep computational models and cadavers that the Injectrode-augmented EBGS therapy increased electric field strength at the fracture site by ~ 70% and 60% respectively compared to conventional EBGS therapy. Rats^27,28^, rabbit^29,30^, and sheep^31^ have been used as animal models in published fracture healing studies investigating electrical stimulation to improve osteogenesis. We intend to use the large-animal sheep model in our study and so focused on studying sheep alongside humans in this study.

## Conclusion

In a large-animal fracture model, sheep, and human, both modeling and cadaver measurements demonstrate that noninvasive EBGS therapy applied transcutaneously generates weak electric fields at the fracture site. These data suggest it is critical to consider how the electric fields applied by stimulation electrodes change at a distant portion of tissue when scaling from culture or small animals to large animals or humans. Our findings (1) help explain the inconsistency in translating promising EBGS results showing improved fracture healing in small animal studies to clinical use and (2) lay the groundwork for a future large-animal study to test the use of Injectrode-augmented EBGSs to further aid bone healing by increasing electric field strengths reaching the deep fracture site.

## Supplementary Information

Supplementary Information.

## Data Availability

All data generated or analyzed during this study are included in this published article (and its Supplementary Information files).
