# Supplementary material for: Efficacy of bone stimulators in large-animal models and humans may be limited by weak electric fields reaching fracture
Source: Sci Rep. 2022 Dec 16;12:21798. doi: 10.1038/s41598-022-26215-w (PMC9758190; doi:10.1038/s41598-022-26215-w)
Supplement: Supplementary file 1 — Supplementary Information. [file 41598_2022_26215_MOESM1_ESM.docx]

# Supplementary Material Table of Contents

Supplementary Material 1 – Electrical conductivity of materials in FEM model

Supplementary Material 2 – Raw waveform from all human cadaver measurements

Supplementary Material 3 – Sensitivity analysis of sheep metatarsus FEM model

**Supplementary Material 1 – Electrical conductivity of materials in FEM model**

**Table 1**. Conductivity of materials in human finite element method (FEM) model

| **Material** | **Origin** | **Conductivity (S/m)** |
| --- | --- | --- |
| Cortical Bone | Human^1^ | 1.0e-2 |
| Bone Marrow (infiltrated) | Bovine^2^ | 2.1e-1 |
| Adipose | Human^2^ | 2.09e-2 |
| Skin | Human^3^ | 8.0e-4 |
| Muscle | Bovine (parallel)^2^ | 2.6e-1 |
| Gel | Hydrogel^4^ | 1.6e-2 |

**Table 2:** Conductivity of materials in sheep FEM model

| **Material** | **Origin** | **Conductivity (S/m)** |
| --- | --- | --- |
| Cortical Bone | Bovine^5^ | 8.0e-3 |
| Bone Marrow | Bovine^2^ | 2.95e-3 |
| Fat | Bovine^2^ | 2.62e-2 |
| Skin | Human^3^ | 8.0e-4 |
| Gel | Hydrogel^4^ | 1.6e-2 |
| Extracellular fluid | Human (heart)^6^ | 6.67e-1 |
| Hematoma | Human (brain)^7^ | 2.0 |
| Articular cartilage | Bovine^8^ | 8.8e-1 |
| Collagen | Bovine (achilles tendon)^9^ | 2e-3 |

**Supplementary Material 2 – Raw waveform from all human cadaver measurements**

(a)


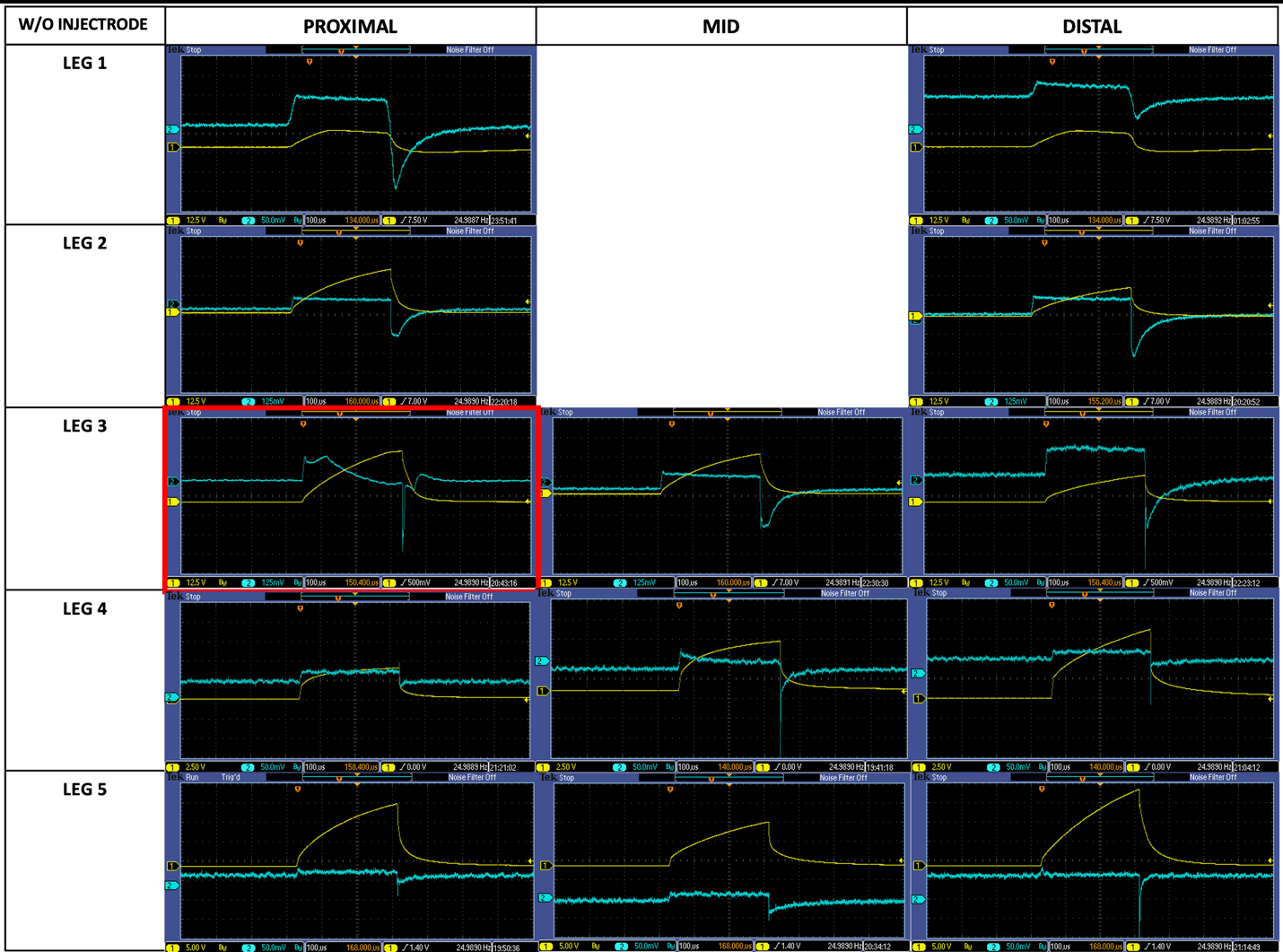


(b)


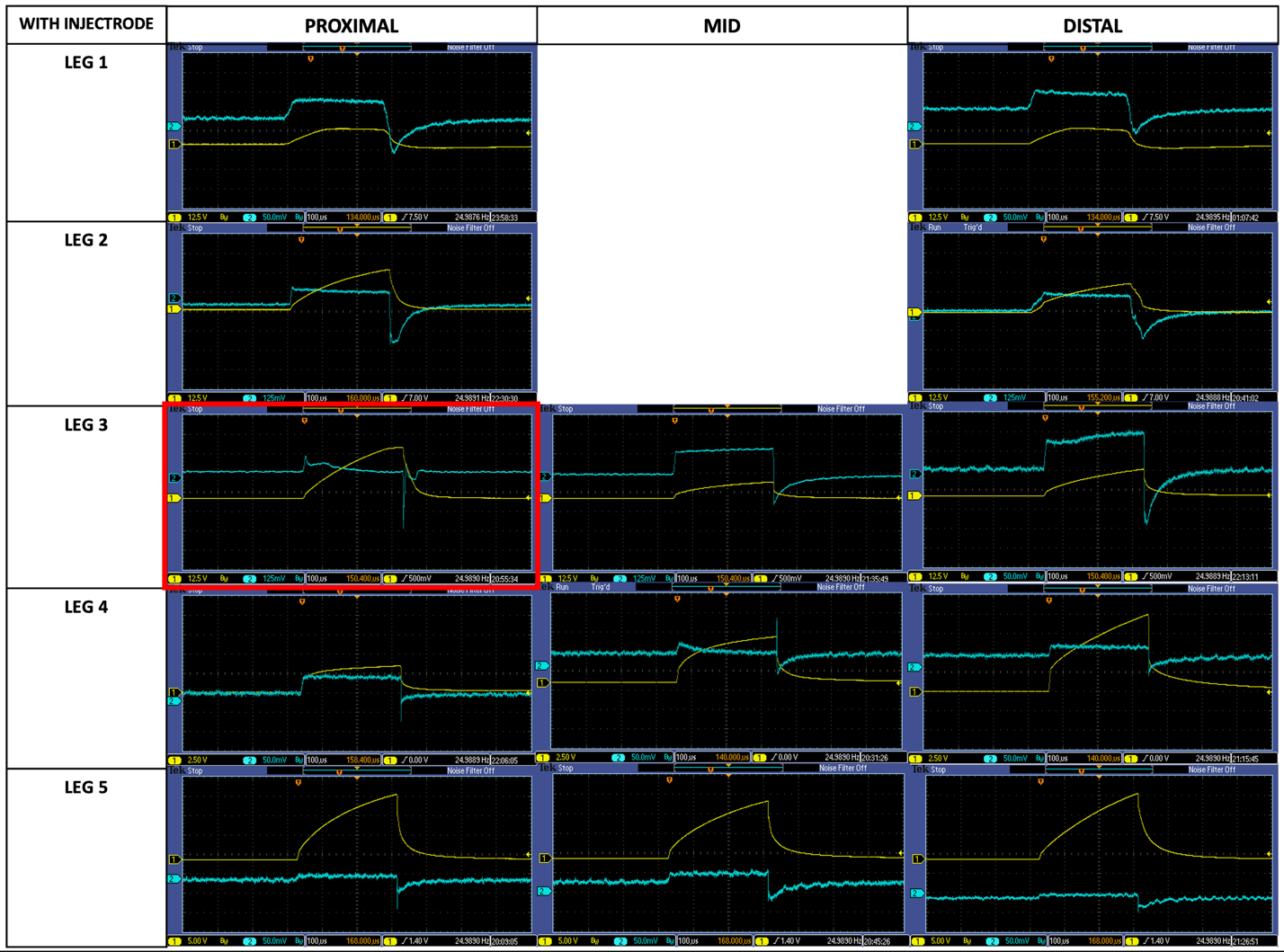


**Figure S2-1:** For human cadaveric modeling, oscilloscope readings were taken for all samples without Injectrode (a) and after Injectrode (b) administration. The average gain was calculated using a total of 12 measurements: 2 from leg 1 (proximal and distal), 2 from Leg 2 (proximal and distal), 2 from Leg 3 (mid and distal), 3 from Leg 4 (proximal, mid, and distal), and 3 from leg 5 (proximal, mid, and distal). Leg 1 and Leg 2 were limited by anatomical distance, making three experimental set-ups on the leg infeasible. The condition outlined in red (proximal cut of Leg 3) was a statistical outlier when comparing gain values. Furthermore, qualitatively, the blue curve (voltage at fracture site) shows an anomaly in the initial slope upward that has a curvilinear descend after. Any other curve that has a descend is either more gradual or more linear. The sharp peak with curved decline indicates a potential anomaly in the bone space that was not visualized or accounted for in the initial cadaveric acquisition. It could also indicate poor electrical contact between the recording electrodes and the bone.

**Supplementary Material 3 – Sensitivity analysis of sheep metatarsus FEM model**

A sensitivity analysis was performed on the sheep metatarsus FEM model. Tissue conductivity and dimensions were varied by 25% above and below their original value and the ratio of electric field at the fracture to the externally applied electric field was measured. We found skin conductivity to be the most sensitive parameter amongst skin, fat, cortical bone, and bone marrow conductivity. In dimensions, we also found skin thickness to be the most sensitive parameter amongst skin thickness, fat thickness, and bone marrow radius. Further, skin conductivity has the most variation in reported values in literature, compared to other parameters, further exasperating the problem of defining an appropriate skin conductivity in the FEM model^4^.

**
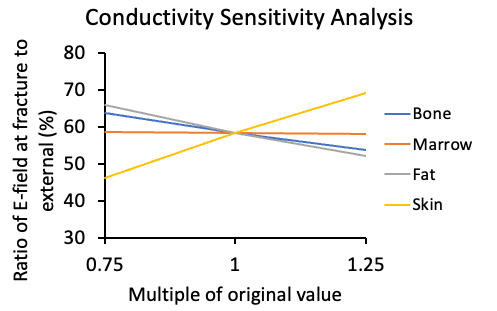
**

**Figure S3-1:** Sheep metatarsus FEM model sensitivity analysis on tissue conductivities showed skin conductivity is the most sensitive.


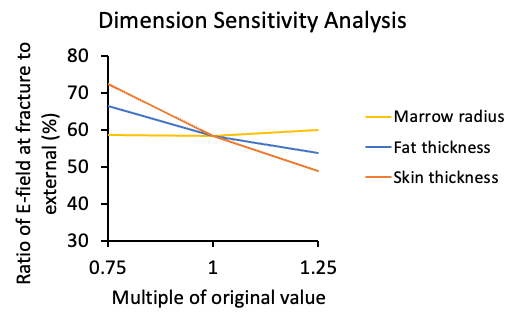


**Figure S3-2:** Sheep metatarsus FEM model sensitivity analysis on tissue dimensions showed skin thickness is the most sensitive.

# Supplementary Material References

1. Kosterich, J. D., Foster, K. R. & Pollack, S. R. Dielectric Permittivity and Electrical Conductivity of Fluid Saturated Bone. *IEEE Trans. Biomed. Eng.* **BME-30**, 81–86 (1983).

2. Gabriel, C. *Compilation of the Dielectric Properties of Body Tissues at RF and Microwave Frequencies.:* http://www.dtic.mil/docs/citations/ADA303903 (1996) doi:10.21236/ADA303903.

3. Raicu, V., Kitagawa, N. & Irimajiri, A. A quantitative approach to the dielectric properties of the skin. *Phys. Med. Biol.* **45**, L1–L4 (2000).

1. Verma, N. *et al.* Augmented Transcutaneous Stimulation Using an Injectable Electrode: A Computational Study. *Front. Bioeng. Biotechnol.* **9**, 796042 (2021).

5. Balmer, T. W., Vesztergom, S., Broekmann, P., Stahel, A. & Büchler, P. Characterization of the electrical conductivity of bone and its correlation to osseous structure. *Sci. Rep.* **8**, 8601 (2018).

6. Spach, M. S., Miller, W. T., Miller-Jones, E., Warren, R. B. & Barr, R. C. Extracellular potentials related to intracellular action potentials during impulse conduction in anisotropic canine cardiac muscle. *Circ. Res.* **45**, 188–204 (1979).

7. Ke, L., Zu, W., Du, Q., Chen, J. & Ding, X. A bio-impedance quantitative method based on magnetic induction tomography for intracranial hematoma. *Med. Biol. Eng. Comput.* **58**, 857–869 (2020).

8. Binette, J. S., Garon, M., Savard, P., McKee, M. D. & Buschmann, M. D. Tetrapolar Measurement of Electrical Conductivity and Thickness of Articular Cartilage. *J. Biomech. Eng.* **126**, 475–484 (2004).

9. Bardelmeyer, G. H. Electrical conduction in hydrated collagen. I. Conductivity mechanisms. *Biopolymers* **12**, 2289–2302 (1973).
